# Solid‐State Janus Nanoprecipitation Enables Amorphous‐Like Heat Conduction in Crystalline Mg_3_Sb_2_‐Based Thermoelectric Materials

**DOI:** 10.1002/advs.202202594

**Published:** 2022-07-18

**Authors:** Rui Shu, Zhijia Han, Anna Elsukova, Yongbin Zhu, Peng Qin, Feng Jiang, Jun Lu, Per O. Å. Persson, Justinas Palisaitis, Arnaud le Febvrier, Wenqing Zhang, Oana Cojocaru‐Mirédin, Yuan Yu, Per Eklund, Weishu Liu

**Affiliations:** ^1^ Department of Materials Science and Engineering Southern University of Science and Technology Shenzhen 518055 China; ^2^ Thin Film Physics Division Department of Physics Chemistry and Biology (IFM) Linköping University Linköping SE‐581 83 Sweden; ^3^ Department of Physics Southern University of Science and Technology Shenzhen 518055 China; ^4^ I. Physikalisches Institut (IA) RWTH Aachen University Sommerfeldstraße14 52074 Aachen Germany; ^5^ Guangdong Provincial Key Laboratory of Functional Oxide Materials and Devices Southern University of Science and Technology Shenzhen Guangdong 518055 China

**Keywords:** atom probe tomography, Janus nanoprecipitation, low thermal conductivity, Mg_3_Sb_2_, thermalelectrics

## Abstract

Solid‐state precipitation can be used to tailor material properties, ranging from ferromagnets and catalysts to mechanical strengthening and energy storage. Thermoelectric properties can be modified by precipitation to enhance phonon scattering while retaining charge‐carrier transmission. Here, unconventional Janus‐type nanoprecipitates are uncovered in Mg_3_Sb_1.5_Bi_0.5_ formed by side‐by‐side Bi‐ and Ge‐rich appendages, in contrast to separate nanoprecipitate formation. These Janus nanoprecipitates result from local comelting of Bi and Ge during sintering, enabling an amorphous‐like lattice thermal conductivity. A precipitate size effect on phonon scattering is observed due to the balance between alloy‐disorder and nanoprecipitate scattering. The thermoelectric figure‐of‐merit ZT reaches 0.6 near room temperature and 1.6 at 773 K. The Janus nanoprecipitation can be introduced into other materials and may act as a general property‐tailoring mechanism.

## Graphical abstract

Bi‐/Ge‐rich Janus nanoprecipitates in Mg_3_Sb_1.5_Bi_0.5_ compounds are uncovered by electron microscopy and atom probe tomography. This complex Janus nanoprecipitate results from local comelting of Bi and Ge during sintering and enables an amorphous‐like lattice thermal conductivity near room temperature. The mechanistic understanding of thermal‐conductivity reduction is supported by modelling the material systems with and without precipitates.

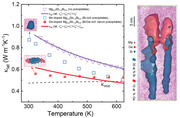

## 1 Introduction

Tailoring thermal conductivity is important for the design of a wide range of materials. Electronic components or high‐temperature protective coatings require efficient heat dissipation. In contrast, thermoelectric materials require low thermal conductivity to achieve high conversion efficiency in waste‐heat harvesting and environmentally friendly cooling.^[^ ^1^ ^]^ The efficiency of thermoelectric materials is determined by the dimensionless figure of merit, defined as *ZT* = *S* ^2^ *σT*/(*κ*_e_+*κ*_lat_), where *S*, *σ*, *T*, *κ*_e_, and *κ*_lat_ are the Seebeck coefficient, electrical conductivity, absolute temperature, charge‐carrier thermal conductivity, and lattice thermal conductivity, respectively. The search for high‐*ZT* materials has drastically progressed since the 1990s by introducing defects and/or reducing dimensionality, aiming at increasing the power factor (*S* ^2^ *σ*) and decreasing *κ*_lat_. Typical approaches include employing atomic‐level disorder,^[^ ^2^ ^]^ dislocations,^[^ ^3^ ^]^ and nanoprecipitates^[^ ^4^ ^]^ to introduce different scattering effects of the confined interfaces on carriers and phonons. In particular, introducing secondary‐phase precipitates over multiple length scales is a general strategy for reducing heat transport by scattering phonons.^[^ ^5^ ^]^

The “nanoparticle‐in‐alloy” concept has been demonstrated as an effective scattering mechanism for mid‐to‐low‐frequency phonons.^[^ ^6^ ^]^ In thermoelectric materials, these nanoparticles are often formed by either precipitation in a homogenous and supersaturated solid solution, or external inclusion during encapsulation. However, in most cases, only single‐type isolated precipitates are obtained.^[^ ^5^, ^7^ ^]^ Recently, side‐by‐side coprecipitation evidenced by atom probe tomography (APT) has been shown to improve the mechanical properties of low‐carbon steels.^[^ ^8^ ^]^ The introduction of such coprecipitation with different sizes, mass, and shapes for each part of the coprecipitates, as well as complex interfaces with the matrix, could enhance the phonon scattering. Conventional nanoprecipitation phonon scattering strategies rely on controlling the number density of precipitates. Yet, theoretical modeling has shown that particle size is also of prominent importance. For example, an optimized silicide particle size of ≈5 nm in SiGe,^[^ ^6^ ^]^ and ≈10 nm ErAs in In_0.53_Ga_0.47_As^[^ ^4^ ^]^ can maximize the phonon scattering by balancing the short‐ and long‐wavelength limit of the scattering cross section.^[^ ^6^ ^]^ However, controlling the optimized size distribution experimentally remains challenging.

Here, we choose Mg_3_(Sb,Bi)_2_‐based materials for investigation. They are promising n‐type thermoelectric compounds with performance comparable to the benchmark n‐type Bi_2_Te_3−_ *_x_* Se*_x_* but without scarce tellurium.^[^ ^9^, ^10^ ^]^ The thermoelectric properties of Mg_3_(Sb,Bi)_2_ can be improved by tuning carrier scattering through chalcogen^[^ ^11^, ^12^ ^]^ or transition metal doping,^[^ ^13^, ^14^ ^]^ as well as by vacancy^[^ ^15^, ^16^ ^]^ and grain boundary engineering.^[^ ^17^, ^18^ ^]^ Near room temperature, the power factor of Mg_3_(Sb,Bi)_2_ has thus been increased to ≈35 µW cm^−1^ K^−2^_,_^[^ ^19^ ^]^ and ZT can be largely improved if eliminating the grain boundary resistance,^[^ ^20^ ^]^ which is promising for room‐temperature thermoelectric applications.^[^ ^9^, ^10^, ^21^, ^22^, ^23^, ^24^ ^]^ Recent theoretical studies uncover that, compared to other Zintl compounds, the enhanced phonon–phonon scattering in Mg_3_Sb_2_‐based materials due to soft Mg bonds,^[^ ^25^ ^]^ locally asymmetric vibrations,^[^ ^26^ ^]^ and high anharmonicity,^[^ ^27^ ^]^ causes an inherently low *κ*_lat_. However, the thermal conductivity of Mg_3_(Sb,Bi)_2_ is still much higher than its amorphous limit^[^ ^28^ ^]^ and thus has the potential to be further reduced through introducing additional phonon scattering mechanisms such as alloying and precipitates, in particular near room temperature. The lattice thermal conductivity (*κ*_lat_) near room temperature is ≈0.93 W m^−1^ K^−1^ for undoped Mg_3_(Sb,Bi)_2_^[^ ^29^ ^]^ and can be further reduced to ≈0.7 W m^−1^ K^−1^ by doping Nb, Co, or Y at the Mg‐sublattice.^[^ ^13^, ^14^, ^30^ ^]^ The reduction of lattice thermal conductivity by the formation of Bi‐rich precipitates has also been reported.^[^ ^9^, ^31^ ^]^ Yet, the critical issues of precipitation kinetics and diversity remain unexplored. Engineering the density and composition of nanoprecipitates in Mg_3_(Sb,Bi)_2_ can therefore demonstrate a reduction in lattice thermal conductivity and a general mechanism for structural tailoring of nanoprecipitates.

## 2 Results and Discussion

### 2.1 Morphology of Janus Nanoprecipitates

**Figure 1:**
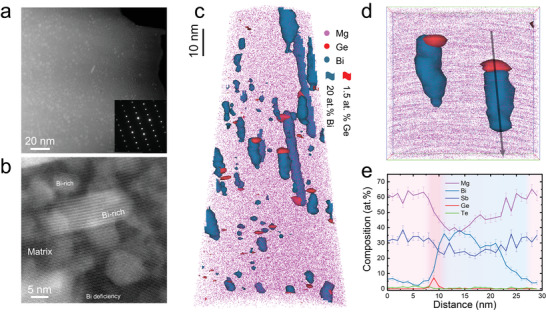
Microstructure of Mg_3.2_Sb_1.47_Bi_0.5_Te_0.02_Ge_0.01_ specimen (Ge‐0.01). a,b) STEM image, revealing a high density of nanoscale precipitates. The insets show the SAED pattern of the corresponding microstructure along the <100> zone axis. c) APT reconstructions showing the elemental distribution (Mg, pink; Ge, red; Bi, teal, Sb and Te atoms are omitted, for clarity). d) Close‐up of a subregion from c, highlighting the 3D structure of Bi/Ge‐rich Janus nanoprecipitates. The dimension of the cuboid region of interest is 30 × 30 × 10 nm^3^. e) 1D composition profile along the arrow in (d).

We have introduced high‐density Bi/Ge‐rich Janus nanoprecipitates in Mg_3.2_(Sb,Bi)_2_ through a local comelting strategy during sintering. The Mg_3.2_(Sb,Bi)_2_ samples were doped with Te and Ge in the composition range from *x* = 0 to 0.05 in steps of 0.01, following the nominal formula of Mg_3.2_Sb_1.49−2_ *_x_* Bi_0.5_Te_0.01+_ *_x_* Ge*_x_*, where Te acts as an effective n‐type dopant to control the carrier concentration.^[^ ^11^ ^]^ The structure of the Mg_3.2_Sb_1.47_Bi_0.5_Te_0.02_Ge_0.01_ (Ge‐0.01) specimen was investigated by scanning transmission electron microscopy (STEM) and APT. **Figure** **1**a shows a high density of ultrafine precipitates (4–16 nm, brighter areas) uniformly distributed within grains. The selected area electron diffraction (SAED) pattern (inset in Figure 1a, <100> zone axis) exhibits an inverse *α*‐La_2_O_3_ trigonal crystal structure of Mg_3_(Sb,Bi)_2_ without additional diffraction spots, indicating that the precipitates have the same crystal structure and coherent interfaces with the matrix. A magnified image (Figure 1b) reveals significant intensity fluctuations, which can be ascribed to compositional fluctuations in the Mg_3_Sb_1.5_Bi_0.5_ matrix given that the high‐angle annular dark field (HAADF) imaging contrast is proportional to ≈*Z* ^2^ (where *Z* is the atomic number). Bi‐rich precipitates show bright contrast due to their large atomic number. Similar Bi‐rich nanoprecipitates were also observed in Mn‐doped Mg_3.2_Sb_1.5_Bi_0.5_,^[^ ^9^, ^31^ ^]^ but not in the Ge‐free Mg_3.2_Sb_1.5_Bi_0.5_ sample (Figure S1, Supporting Information). This kind of local compositional fluctuations could result from the non‐equilibrium synthesis route, i.e., ball milling and hot pressing. However, the number density of the Bi‐rich nanoprecipitates in the as‐fabricated Ge‐doped Mg_3.2_Sb_1.5_Bi_0.5_ is much higher than that in Mn‐doped Mg_3.2_Sb_1.5_Bi_0.5_.^[^ ^9^ ^]^ To explore the fundamental mechanism, we conducted APT characterization, which constructs the 3D distribution of constituent elements with sub‐nanometer spatial resolution and tens of parts‐per‐million elemental sensitivity.^[^ ^32^ ^]^ Figure 1c shows an APT reconstruction of the Ge‐0.01 sample, confirming the presence of a large volume fraction of Bi‐rich precipitates highlighted by the iso‐composition surface of 20 at% Bi. The size of Bi‐rich precipitates ranges between 2 and 20 nm in the APT reconstruction and is consistent with STEM observations (Figure 1d). The number density of Bi‐rich precipitates is estimated to be 3.0 × 10^23^ m^−3^. Remarkably, a similarly high number of Ge‐rich precipitates (0.9 × 10^23^ m^−3^), as depicted by the iso‐composition surface of 1.5 at% Ge, are found to connect side‐by‐side with the Bi‐rich precipitates, forming Janus particles, i.e., particles composed of two different phases on either side.^[^ ^33^ ^]^ A close‐up of the 3D morphology of Ge/Bi‐rich Janus precipitates is shown in Figure 1d. The composition profile across the Janus particle in Figure 1e as indicated by the arrow in Figure 1d reveals an average composition of 28.6 ± 1.5 at% Bi for Bi‐rich precipitates, and 8.5 ± 1.5 at% Ge for Ge‐rich precipitates, which are much higher than the corresponding values of 9.5 ± 1.5 at% Bi and 1.5 ± 1.0 at% Ge in the matrix.

**Figure 2:**
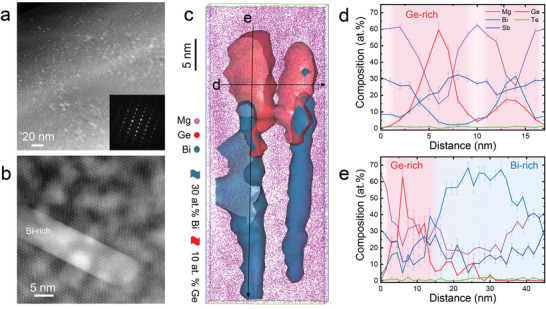
Microstructure of the Mg_3.2_Sb_1.39_Bi_0.5_Te_0.06_Ge_0.05_ specimen (Ge‐0.05). a) STEM image, revealing high‐density nanoprecipitates. The insets show the corresponding SAED pattern. b) Magnified view of a Bi‐rich precipitate. c) APT reconstructions showing the elemental distribution (Mg, pink; Ge, red; Bi, teal); the Ge‐rich and Bi‐rich precipitates are depicted by iso‐composition surfaces of 10 at% Ge and 30 at% Bi, respectively. Both precipitates connect side‐by‐side forming Janus precipitates. d) 1D composition profile calculated along the horizontal arrow across two Ge‐rich precipitates. e) 1D composition profile calculated along the vertical arrow across the Janus precipitate from Ge‐rich to Bi‐rich part.

We further investigated the corresponding microstructure of Mg_3.2_Sb_1.39_Bi_0.5_Te_0.06_Ge_0.05_ (Ge‐0.05). The Janus nanoprecipitates also exist in a large volume fraction but are coarser than that in the sample with lower Ge contents. STEM images in **Figure** **2**a,b show elongated nanoprecipitates in size of 5–50 nm embedded in the matrix (inset in Figure 2a, <100> zone axis, and X‐ray diffraction (XRD), see Figure S2, Supporting Information). Furthermore, Figure 2c shows two Bi/Ge rich Janus nanoprecipitates with a size of ≈50 nm in length and ≈5 nm in diameter depicted by the iso‐composition surface of 30 at% Bi and 10 at% Ge. The side‐by‐side configuration consolidates the unique Janus feature in the as‐fabricated Ge‐doped Mg_3.2_Sb_1.5_Bi_0.5_. Note that two types of Bi‐rich precipitates, i.e., Bi‐rich Mg_3_(Bi,Sb)_2_ phase and pure Bi phase, are observed in the high Ge doping sample, which is confirmed by the STEM phase identification (Figures S3 and S4, Supporting Information). The Ge‐rich particle has been observed by STEM after in situ annealing treatment at 573 K and is confirmed with the same structure as the matrix (Figure 2a; Figures S5 and S6, Supporting Information), i.e., Mg_3_(Bi,Sb)_2_ phase, but with high Ge content to 9 at%.

Both Bi‐ and Ge‐rich parts of the Janus nanoprecipitates coarsen with increasing Ge content. Based on the APT analysis, the number density of precipitates decreases from 3.0 × 10^23^ m^−3^ (Ge‐0.01 sample) to 6.4 × 10^22^ m^−3^ (Ge‐0.05 sample). The Ge content gradually decreases from the core of precipitates to the matrix, as revealed by the 1D composition profile across the Ge‐rich precipitates (Figure 2d). The maximum composition of about 60 at% Ge in the precipitate core is higher than that for the lower Ge‐content sample (Figure 1e) due to the coarsening of precipitates with increasing Ge content. The content of Bi in the Bi‐rich precipitates is also increased as determined by the 1D composition profile across the Janus particle from the Ge‐rich part to the Bi‐rich part (Figure 2e). Furthermore, in addition to the Ge–Bi configuration, we observed Ge‐Bi‐Ge precipitates, especially in samples with high Ge contents (Figure S7, Supporting Information).

### 2.2 Kinetic Mechanism of Janus Nanoprecipitation

**Figure 3:**
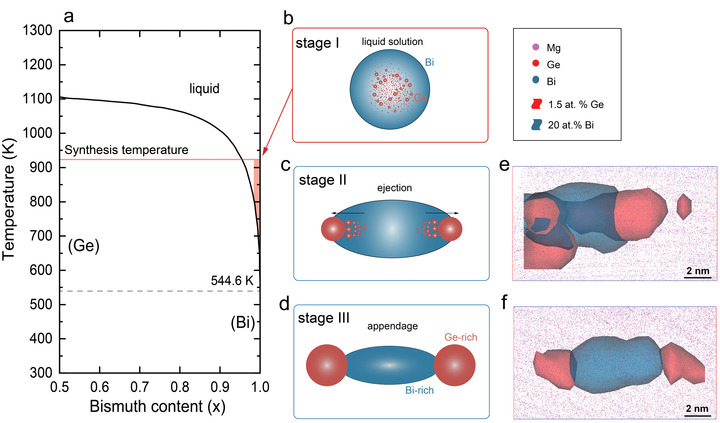
Mechanism of Janus precipitation. a) Bi‐Ge binary phase diagram.^[^ ^34^ ^]^ Red‐colored areas denote the Ge‐doping limitation for Mg_3.2_Sb_1.5_Bi_0.5_ compounds. b–d) Schematic plots of the co‐precipitation process during sintering and cooling, which can be divided into 3 stages. e,f) Experimental observation from APT reconstructions for correlative stage II, and III.

The addition of Ge plays a critical role in the formation of Bi/Ge‐rich Janus nanoprecipitation in the as‐fabricated Mg_3.2_(Sb,Bi)_2_. **Figure** **3**a shows the Bi‐Ge binary phase diagram,^[^ ^34^ ^]^ with a colored area denoting the Ge‐doping region in this work. The mutual solid‐state solubilities of Ge and Bi are negligible, but Ge and Bi are fully miscible at the sintering temperature of 923 K. The formation of Bi/Ge‐rich Janus nanoprecipitation could be explained by a co‐melting mechanism. The as‐fabricated Mg_3.2_(Sb,Bi)_2_ is made through a powder metallurgy route with high‐energy ball milling (BM) followed by spark plasma sintering (SPS), which provides the energy necessary for the atomic diffusion into a crystalline structure at a temperature below the melting point of Mg_3_(Sb,Bi)_2_. Here, the sintering temperature is 923 K, higher than the melting point (544.6 K) of Bi. The formation of liquid Bi could be competitive with the crystallization of Mg_3_(Sb,Bi)_2_. At the first stage (Figure 3b), Bi forms a local liquid phase providing a molten reservoir for the Ge (melting point: 1211.5 K). The solubility of Ge in the solid Bi matrix is negligible, while it is 0.95% in liquid Bi at 923 K. Without Ge, the local liquid Bi will be consumed by the crystallization of the Mg_3_(Sb,Bi)_2_ from the amorphous‐like ball‐milled powders. The crystallization of Mg_3_(Sb,Bi)_2_ and Bi‐Ge liquid phase compete with each other. The addition of Ge shifts the balance of the two processes, resulting in the local Bi‐Ge liquid phase remaining until the cooling process, in which the local Bi‐Ge liquid phase is quickly encapsulated in Mg_3_(Sb,Bi)_2_ crystalline phase. At the second stage (Figure 3c), corresponding to a cooling process, the solute Ge starts precipitating and is ejected to the sides of the local Bi‐rich liquid phase region. The Ge atoms do not simply thicken the shell, but rather form Ge appendages attached to the sides of the Bi‐rich part. It can be explained from the view of thermodynamics and kinetics. The free energy reduction of the Ge‐rich phase heterogeneously nucleated on Bi‐rich precipitate exceeds the energy penalty of a Bi‐/Ge‐rich interface, and the insoluble Ge atoms diffuse in Bi‐rich precipitates along <*a*, *b*> direction much easier than *c* direction. At the third stage (Figure 3d), corresponding to cooling down to the melting point of Bi, the local Bi‐rich liquid phase solidifies, finally leading to a high density of Janus precipitates. Residual microstructural evidence of these two stages was observed in the medium Ge‐doped sample (Ge‐0.03, Figure S7, Supporting Information), as shown in the APT reconstructions in Figure 3e,f. Generally, the addition of Ge plays a vital role in preventing the liquid Bi from being consumed by the crystallization of Mg_3_(Sb,Bi)_2_, resulting in the final Bi/Ge‐rich Janus nanoprecipitation. From the viewpoint of thermodynamics, the contribution of the configuration entropy makes the Bi–Ge liquid phase more stable than the pure Bi liquid. Higher Ge content leads to a larger volume of local Bi–Ge liquid phase and finally larger size of Bi/Ge‐rich Janus nanoprecipitation in the bulk materials.

### 2.3 Reduction of *κ*_lat_ and Enhancement of *ZT*

**Figure 4:**
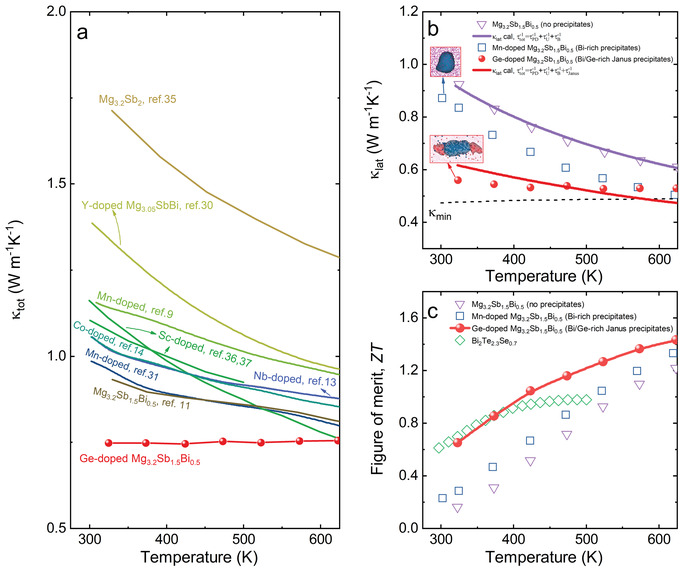
Thermoelectric performance of n‐type Ge‐doped Mg_3.2_Sb_1.5_Bi_0.5_. a) A comparison of total thermal conductivity *κ*_tot_ among several polycrystalline n‐type Mg_3_(Sb,Bi)_2_ materials: Mg_3.2_Sb_2_,^[^ ^35^ ^]^ Mg_3.2_Sb_1.5_Bi_0.5_,^[^ ^11^ ^]^ transition‐metal (Nb,^[^ ^13^ ^]^ Co,^[^ ^14^ ^]^ Mn,^[^ ^9^, ^31^ ^]^ Sc,^[^ ^36^, ^37^ ^]^ Y^[^ ^30^ ^]^) doped Mg_3_(Sb,Bi)_2_, and Janus precipitation Mg_3.2_Sb_0.5_Bi_0.5_. b) Temperature dependence of lattice thermal conductivity *κ*_lat_ of dopant‐free Mg_3.2_Sb_1.5_Bi_0.5_ (no precipitates), Mg_3.15_Mn_0.05_Sb_1.5_Bi_0.5_ [single‐type Bi‐rich precipitates, ref.[31]] and Mg_3.2_Sb_1.47_Bi_0.5_Te_0.02_Ge_0.01_ (Bi/Ge‐rich Janus precipitates). The solid purple and red lines in (b) show the temperature‐dependent lattice thermal conductivity *κ*_lat_ calculated with the Callaway model and input parameters obtained from STEM and APT investigation of Ge‐free and Ge‐0.01 doping samples. c) Figure of merit, *ZT* of above three Mg_3.2_Sb_1.5_Bi_0.5_‐based samples in comparison with state‐of‐the‐art n‐type thermoelectric material Bi_2_Te_2.3_Se_0.7_.^[^ ^38^ ^]^

**Figure** **4**a shows temperature‐dependent thermal conductivity of as‐fabricated Ge‐doped Mg_3.2_Sb_1.5_Bi_0.5_ with Janus precipitations, compared with reported n‐type Mg_3_Sb_2_‐based materials.^[^ ^9^, ^11^, ^13^, ^14^, ^30^, ^31^, ^35^, ^36^, ^37^ ^]^ The as‐fabricated Mg_3.2_Sb_1.5_Bi_0.5_ with Janus precipitates shows strikingly low and amorphous‐like thermal conductivity values. We compared the lattice thermal conductivity of the as‐fabricated pristine Mg_3.2_Sb_1.5_Bi_0.5_, Mg_3.2_Sb_1.5_Bi_0.5_ with single Bi‐rich precipitate induced by Mn dopant, and Mg_3.2_Sb_1.5_Bi_0.5_ with Bi/Ge Janus nanoprecipitate in Figure 4b. The electrical transport properties of these three samples are shown in Figure S8 in the Supporting Information). The lattice thermal conductivity (*κ*_lat_) was deduced from the subtraction of the total thermal conductivity by the electronic thermal conductivity (*κ*_e_) calculated from the Wiedemann–Franz law, *κ*_e_ = *LσT* with the temperature dependent Lorenz number *L* derived from the Seebeck coefficient.^[^ ^39^ ^]^ The *κ*_lat_ of Mg_3.2_Sb_1.5_Bi_0.5_ is significantly decreased due to the strong phonon scattering by the Bi/Ge Janus nanoprecipitates, especially in the temperature range of 300–625 K. It is well known that the temperature‐dependent *κ*_lat_ of a normal crystal with an acoustic‐phonon‐dominated scattering mechanism follows an inverse power law, i.e., *κ*_lat_∝*T* ^−1^. However, the temperature‐dependent *κ*_lat_ of the Janus nanoprecipitate systems strongly deviates from the relationship of *κ*_lat_∝*T* ^−1^, and approaches the amorphous limit *κ*_lat_∝*T* ^0^. A comparison of the exponent value (*r*) in the *κ*_lat_∝*T ^r^* relation between the pristine Mg_3.2_Sb_1.5_Bi_0.5_ and the Janus‐nanostructured Mg_3.2_Sb_1.5_Bi_0.5_ in Figure 4b shows that the Janus‐nanostructured Mg_3.2_Sb_1.5_Bi_0.5_ has an exponent value of *r* = −0.1, which approaches the amorphous limit of Mg_3.2_Sb_1.5_Bi_0.5_ determined using the Cahill model^[^ ^40^ ^]^ (see the Supporting Information). Although high‐density nanoprecipitates are demonstrated to lower the lattice thermal conductivity for many thermoelectric materials, such as PbTe, this amorphous‐like behavior has not been achieved through regular precipitation.

It is well known that phonons have a wider distribution in the mean free path than that of electrons.^[^ ^41^ ^]^ In a classic treatment of the phonon scattering of the nanoparticles, the relaxation time is only related to the particle size.^[^ ^42^ ^]^ Mingo et al.^[^ ^6^ ^]^ has theoretically shown a size‐dependent *κ*_lat_ in SiGe with silicide nanoparticles, suggesting an optimized particle size, but lacking direct observation. In this Janus‐nanostructured Mg_3.2_Sb_1.5_Bi_0.5_, the size of Bi/Ge Janus nanoprecipitates could be tuned by different Ge contents and a size of ≈11 nm for Bi‐rich precipitates can most efficiently scatter the phonons (Figure S9, Supporting Information). Furthermore, the calculated lattice thermal conductivity is provided, based on the Callaway model^[^ ^43^ ^]^ and parameters from the STEM and APT investigations (Section S8, Supporting Information). The phonon scattering of the Janus particles could be considered as a combination of an average effect of the whole particle and partial contributions of the internal sub‐nanoparticle. Considering that the matrix is a heavily doped system with spherical nanoparticles, we only consider the Rayleigh limit (*σ*_l_). The frequency‐dependent phonon relaxation time of Janus nanoparticles (*τ*_Jnp_) can be derived as

(1) $$\begin{array}{ccc} \frac{1}{\tau_{\mathrm{jnp}}} & = & \frac{\upsilon }{3}\frac{f_{\langle \mathrm{jnp}\rangle }}{R_{\langle \mathrm{jnp}\rangle }}{\left(\frac{D_{\langle \mathrm{jnp}\rangle }-D_{0}}{D_{0}}\right)}^{2}{\left(\frac{\omega }{\upsilon }R_{\langle \mathrm{jnp}\rangle }\right)}^{4}\\ & & +\;\sum_{i}\frac{1}{3{\left(m\upsilon \right)}^{3}}\frac{f_{i-\mathrm{np}}}{R_{i-\mathrm{np}}}{\left(\frac{D_{i-\mathrm{np}}-D_{\langle \mathrm{jnp}\rangle }}{D_{\langle \mathrm{jnp}\rangle }}\right)}^{2}{\left(\omega R_{i-\mathrm{np}}\right)}^{4} \end{array}$$

where *f*_jnp_, *R*_jnp_, *D*_jnp_ are the average volume fraction, radius, and density of the Janus nanoparticle, respectively, *D*_0_ is the density of the matrix, *υ* is the average phonon speed, *ω* is the angular frequency, and m is a correction factor. The first term of the right side of Equation (1) considers the Janus particle as an entirety, while the second term of the right side of Equation (1) summarizes all the subparticles within the Janus particle. In principle, the Janus particle provides a wide frequency range of phonon scattering. The calculated *κ*_lat_ for Ge‐free Mg_3.2_Sb_1.5_Bi_0.5_ matches the experimental data well as a function of temperature, and that for Mg_3.2_Sb_1.47_Bi_0.5_Te_0.02_Ge_0.01_ with Janus nanoprecipitates is reasonably close to experimental data but has a stronger temperature dependence. It indicates that the Janus precipitate structure increases the frequency range for phonon scattering, and hence significantly reduces *κ*_lat_ near room temperature. The thermoelectric properties of Mg_3.2_(Sb,Bi)_2_ with different contents of Ge dopant were characterized. An increased thermoelectric figure‐of‐merit *ZT* = 0.6 near room temperature (Figure 4c) and 1.6 at 773 K are achieved, corresponding to an enhancement of 170% at room temperature and 8% at 773 K, respectively, compared with the Ge‐free sample (Figure S10, Supporting Information).

## 3 Conclusion

In summary, we report a strategy to tune thermal conductivity by engineering the hierarchical structural aspects of nanoprecipitates, including composition, phase, and morphology. In the as‐fabricated polycrystalline bulk Ge‐doped Mg_3.2_Sb_1.5_Bi_0.5_, a local comelting strategy between Bi and Ge induces the formation of Janus nanoprecipitates. Two distinct nanoprecipitates with different masses provide a way to scatter a broader frequency range of phonons compared with individual precipitate structures, resulting in enhanced thermoelectric properties at low and intermediate temperatures for n‐type Mg_3.2_Sb_1.5_Bi_0.5_. This liquid‐encapsulation‐induced Janus precipitation approach is expected to be applicable to other thermoelectric materials and more generally to reduce thermal conductivity. We have provided a simple model to address the phonon scattering from both the average effect of the whole particle and partial contributions of internal sub‐nanoparticles. This work thus provides a new perspective for tailoring hierarchical structures of nanoinclusions for low thermal conductivity and thermoelectric properties.

## 4 Experimental Section

### Materials Synthesis

High purity magnesium turnings (Mg, 99.8%; Alfa Aesar), antimony shots (Sb, 99.999%; 5N Plus), bismuth shots (Bi, 99.999%; 5N Plus), tellurium shots (Te, 99.999%; 5N Plus), and germanium powders (Ge, 99.99%; Alfa Aesar) were weighed according to the stoichiometric composition of Mg_3.2_Bi_0.5_Sb_1.49−2_ *_x_* Te_0.01+_ *_x_* Ge*_x_* (*x* = 0, 0.006, 0.01, 0.02, 0.03, 0.04, and 0.05). The extra Mg was used for compensating for the loss during synthesis. All the elements were mixed into a stainless‐steel ball milling jar in a glove box under an argon atmosphere with an oxygen level <0.1 ppm. The materials were ball‐milled for 10 h. The ball‐milled powders were then loaded into a graphite die with an inner diameter of 15 mm in a glove box. The graphite die with loaded powder was immediately hot pressed at 923 K for 10 min. The sintering was done in a vacuum atmosphere at a pressure of 50 MPa. The thickness of hot‐pressed disks was about 12 mm.

### Structural Characterization

XRD was carried out on a PANalytical X'Pert powder diffractometer with a Cu source (*λ*_K*α*_ ≈ 1.5406 Å) operated at 45 kV/40 mA. Scanning transmission electron microscopy high angle annular dark field (STEM‐HAADF) imaging and STEM energy‐dispersive X‐ray spectroscopy (STEM‐EDX) analysis, with a Super‐X EDX detector, were performed in the Linköping's monochromated, high‐brightness, double‐corrected FEI Titan^3^, operated at 300 kV. The specimen for TEM examination was prepared by mechanical grinding followed by Ar^+^‐ion milling using a Gatan 691 Precision Ion Polishing Systems at liquid nitrogen temperature.

### Atom Probe Tomography Measurement

Needle‐shaped APT specimens with an apex diameter of about 50 nm for samples Mg_3.2_Bi_0.5_Sb_1.49−2_ *_x_* Te_0.01+_ *_x_* Ge*_x_* were prepared using a dual‐beam focused‐ion beam (SEM/FIB) microscope (FEI Helios 650 NanoLab) equipped with a micromanipulator according to the standard lift‐out method. The last step of the tip sharpening process utilized a low voltage and current (5 kV, 8 pA) Ga^+^ ion beam to minimize Ga implantation in the sample (Ga content of the region analyzed was <0.01 at%). APT experiments were conducted on a Cameca LEAP‐4000X Si equipped with a picosecond UV laser (wavelength 355 nm). The specimen was maintained at 40 K and laser energy of 10 pJ was used at a pulse rate of 200 kHz with a target evaporation rate of 5 ions per 1000 laser pulses. The ion flight path was 160 mm. Ions were detected using a position‐sensitive detector with a detection efficiency of ≈50%. This detection efficiency is the same for all ions evaporated. The data collected were 3D reconstructed and analyzed using the program IVAS v.3.8.0.

### Thermoelectric Characterization

All the samples were cut into about 2.5 mm × 3 mm × 14 mm pieces, which were coated with a thin‐layer BN to protect instruments, to simultaneously measure the electrical resistivity and Seebeck coefficient under a low‐pressure helium atmosphere from RT to 623 K (ZEM‐3; ULVAC Riko). The thermal diffusivity *D* was measured by the laser flash method (Netzsch LFA 467) and the thermal conductivity *κ* was calculated from *κ* = *dDC*_p_, where density (*d*) of the samples was measured by the Archimedean method, the specific heat (*C*_p_) was taken from the previous studies.^[^ ^9^ ^]^ Electrical and thermal transport properties were both measured from the same as‐pressed disk in directions perpendicular to the direction in which the pressure was applied to the samples during synthesis.

## Conflict of Interest

The authors declare no conflict of interest.

## Author Contributions

W.L. and R.S. initiated the study. W.L., P.E., and Y.Y. supervised the research. R.S., Z.H, and P.Q. synthesized samples and performed part of microstructure characterization and thermoelectric property measurements. Y.Z. R.S.F.J., and W.L. performed the theoretical calculations of lattice thermal conductivity in discussion with W.Z.R.S. prepared TEM specimens. A.E., R.S., J.L., and J.P. performed the STEM analysis with contributions from P.O.Å.P., A.le F., and P.E. The APT measurements were iniated by O.C.M. who also contributed to discussion and interpretations. Y.Y. performed APT experiments and data analysis. R.S., W.L., P.E., and Y.Y wrote the manuscript with contributions from the co‐authors. All co‐authors read, edited, and commented on successive version of the manuscript.

## Supporting information

Supporting Information

## Data Availability

The data that support the findings of this study are available from the corresponding authors upon reasonable request.

## References

[1] G. J. Snyder , E. S. Toberer , Nat. Mater. 2008, 7, 105. 18219332 10.1038/nmat2090

[2] B. Jiang , Y. Yu , J. Cui , X. Liu , L. Xie , J. Liao , Q. Zhang , Y. Huang , S. Ning , B. Jia , B. Zhu , S. Bai , L. Chen , S. J. Pennycook , J. He , Science 2021, 371, 830. 33602853 10.1126/science.abe1292

[3] Z. Chen , B. Ge , W. Li , S. Lin , J. Shen , Y. Chang , R. Hanus , G. J. Snyder , Y. Pei , Nat. Commun. 2017, 8, 13828. 28051063 10.1038/ncomms13828 PMC5216132

[4] W. Kim , J. Zide , A. Gossard , D. Klenov , S. Stemmer , A. Shakouri , A. Majumdar , Phys. Rev. Lett. 2006, 96, 045901. 16486849 10.1103/PhysRevLett.96.045901

[5] K. F. Hsu , S. Loo , F. Guo , W. Chen , J. S. Dyck , C. Uher , T. Hogan , E. K. Polychroniadis , M. G. Kanatzidis , Science 2004, 303, 818. 14764873 10.1126/science.1092963

[6] N. Mingo , D. Hauser , N. P. Kobayashi , M. Plissonnier , A. Shakouri , Nano Lett. 2009, 9, 711. 19128146 10.1021/nl8031982

[7] K. Biswas , J. He , I. D. Blum , C.‐I. Wu , T. P. Hogan , D. N. Seidman , V. P. Dravid , M. G. Kanatzidis , Nature 2012, 489, 414. 22996556 10.1038/nature11439

[8] Z. B. Jiao , J. H. Luan , M. K. Miller , Y. W. Chung , C. T. Liu , Mater. Today 2017, 20, 142.

[9] R. Shu , Y. Zhou , Q. Wang , Z. Han , Y. Zhu , Y. Liu , Y. Chen , M. Gu , W. Xu , Y. Wang , W. Zhang , L. Huang , W. Liu , Adv. Funct. Mater. 2019, 29, 1807235.

[10] J. Mao , H. Zhu , Z. Ding , Z. Liu , G. A. Gamage , G. Chen , Z. Ren , Science 2019, 365, 495. 31320557 10.1126/science.aax7792

[11] H. Tamaki , H. K. Sato , T. Kanno , Adv. Mater. 2016, 28, 10182. 27690372 10.1002/adma.201603955

[12] J. Zhang , L. Song , S. H. Pedersen , H. Yin , L. T. Hung , B. B. Iversen , Nat. Commun. 2017, 8, 13901. 28059069 10.1038/ncomms13901 PMC5227096

[13] J. Shuai , J. Mao , S. Song , Q. Zhu , J. Sun , Y. Wang , R. He , J. Zhou , G. Chen , D. J. Singh , Z. Ren , Energy Environ. Sci. 2017, 10, 799.

[14] J. Mao , J. Shuai , S. Song , Y. Wu , R. Dally , J. Zhou , Z. Liu , J. Sun , Q. Zhang , C. dela Cruz , S. Wilson , Y. Pei , D. J. Singh , G. Chen , C.‐W. Chu , Z. Ren , Proc. Natl. Acad. Sci. 2017, 114, 10548. 28923974 10.1073/pnas.1711725114 PMC5635921

[15] J. Shuai , B. Ge , J. Mao , S. Song , Y. Wang , Z. Ren , J. Am. Chem. Soc. 2018, 140, 1910. 29332381 10.1021/jacs.7b12767

[16] J. Mao , Y. Wu , S. Song , Q. Zhu , J. Shuai , Z. Liu , Y. Pei , Z. Ren , ACS Energy Lett. 2017, 2, 2245.

[17] J. J. Kuo , S. D. Kang , K. Imasato , H. Tamaki , S. Ohno , T. Kanno , G. J. Snyder , Energy Environ. Sci. 2018, 11, 429.

[18] T. Luo , J. J. Kuo , K. J. Griffith , K. Imasato , O. Cojocaru‐Mirédin , M. Wuttig , B. Gault , Y. Yu , G. J. Snyder , Adv. Funct. Mater. 2021, 31, 2100258.

[19] Z. Han , Z. Gui , Y. B. Zhu , P. Qin , B.‐P. Zhang , W. Zhang , L. Huang , W. Liu , Research 2020, 2020, 1672051. 32190833 10.34133/2020/1672051 PMC7064820

[20] J. J. Kuo , M. Wood , T. J. Slade , M. G. Kanatzidis , G. J. Snyder , Energy Environ. Sci. 2020, 13, 1250.

[21] X. Shi , C. Sun , Z. Bu , X. Zhang , Y. Wu , S. Lin , W. Li , A. Faghaninia , A. Jain , Y. Pei , Adv. Sci. 2019, 6, 1802286. 10.1002/advs.201802286 PMC6702648 31453051

[22] K. Imasato , S. D. Kang , G. J. Snyder , Energy Environ. Sci. 2019, 12, 965.

[23] M. Wood , J. J. Kuo , K. Imasato , G. J. Snyder , Adv. Mater. 2019, 31, 1902337. 10.1002/adma.201902337 31273874

[24] F. Zhang , C. Chen , H. Yao , F. Bai , L. Yin , X. Li , S. Li , W. Xue , Y. Wang , F. Cao , X. Liu , J. Sui , Q. Zhang , Adv. Funct. Mater. 2020, 30, 1906143.

[25] W. Peng , G. Petretto , G.‐M. Rignanese , G. Hautier , A. Zevalkink , Joule 2018, 2, 1879.

[26] Y. Zhu , Y. Xia , Y. Wang , Y. Sheng , J. Yang , C. Fu , A. Li , T. Zhu , J. Luo , C. Wolverton , G. J. Snyder , J. Liu , W. Zhang , Research 2020, 2020, 4589786. 33623905 10.34133/2020/4589786 PMC7877392

[27] J. Ding , T. Lanigan‐Atkins , M. Calderón‐Cueva , A. Banerjee , D. L. Abernathy , A. Said , A. Zevalkink , O. Delaire , Sci. Adv. 2021, 7, eabg1449. 34020958 10.1126/sciadv.abg1449 PMC8139592

[28] K. Imasato , S. Ohno , S. D. Kang , G. J. Snyder , APL Mater. 2018, 6, 016106.

[29] Y. Pan , M. Yao , X. Hong , Y. Zhu , F. Fan , K. Imasato , Y. He , C. Hess , J. Fink , J. Yang , B. Büchner , C. Fu , G. J. Snyder , C. Felser , Energy Environ. Sci. 2020, 13, 1717.

[30] X. Shi , T. Zhao , X. Zhang , C. Sun , Z. Chen , S. Lin , W. Li , H. Gu , Y. Pei , Adv. Mater. 2019, 31, 1903387. 10.1002/adma.201903387 31276253

[31] X. Chen , H. Wu , J. Cui , Y. Xiao , Y. Zhang , J. He , Y. Chen , J. Cao , W. Cai , S. J. Pennycook , Z. Liu , L.‐D. Zhao , J. Sui , Nano Energy 2018, 52, 246.

[32] Y. Yu , C. Zhou , S. Zhang , M. Zhu , M. Wuttig , C. Scheu , D. Raabe , G. J. Snyder , B. Gault , O. Cojocaru‐Mirédin , Mater. Today 2020, 32, 260.

[33] A. Walther , A. H. E. Müller , Chem. Rev. 2013, 113, 5194. 23557169 10.1021/cr300089t

[34] R. W. Olesinski , G. J. Abbaschian , Bull. Alloy Phase Diagrams 1986, 7, 535.

[35] S. Ohno , K. Imasato , S. Anand , H. Tamaki , S. D. Kang , P. Gorai , H. K. Sato , E. S. Toberer , T. Kanno , G. J. Snyder , Joule 2018, 2, 141.

[36] X. Shi , C. Sun , X. Zhang , Z. Chen , S. Lin , W. Li , Y. Pei , Chem. Mater. 2019, 31, 8987. 10.1002/adma.201903387 31276253

[37] J. Zhang , L. Song , B. B. Iversen , Angew. Chem., Int. Ed. 2020, 59, 4278. 10.1002/anie.201912909 31850591

[38] W.‐S. Liu , Q. Zhang , Y. Lan , S. Chen , X. Yan , Q. Zhang , H. Wang , D. Wang , G. Chen , Z. Ren , Adv. Energy Mater. 2011, 1, 577.

[39] H.‐S. Kim , Z. M. Gibbs , Y. Tang , H. Wang , G. J. Snyder , APL Mater. 2015, 3, 041506.

[40] D. G. Cahill , S. K. Watson , R. O. Pohl , Phys. Rev. B 1992, 46, 6131. 10.1103/physrevb.46.6131 10002297

[41] X. Qian , J. Zhou , G. Chen , Nat. Mater. 2021, 20, 1188. 33686278 10.1038/s41563-021-00918-3

[42] A. Majumdar , J. Heat Transfer 1993, 115, 7.

[43] J. Callaway , H. C. von Baeyer , Phys. Rev. 1960, 120, 1149.

