# Supplementary material for: Solid‐State Janus Nanoprecipitation Enables Amorphous‐Like Heat Conduction in Crystalline Mg3Sb2‐Based Thermoelectric Materials
Source: Adv Sci (Weinh). 2022 Jul 18;9(25):2202594. doi: 10.1002/advs.202202594 (PMC9443448; doi:10.1002/advs.202202594)
Supplement: Supplementary file 1 — Supporting Information [file ADVS-9-2202594-s001.pdf]

Supplementary Materials for

Solid-state Janus nanoprecipitation enables amorphous-like heat conduction in crystalline Mg_3Sb_2-based thermoelectric materials

Rui Shu, Zhijia Han, Anna Elsukova, Yongbin Zhu, Peng Qin, Feng Jiang, Jun Lu, Per O. Å. Persson, Justinas Palisaitis, Arnaud le Febvrier, Wenqing Zhang, Oana Cojocaru-Mirédin, Yuan Yu*, Per Eklund* and Weishu Liu*

R. Shu, Z. Han, Y.B. Zhu, P. Qin, F. Jiang, W. S. Liu
Department of Materials Science and Engineering
Southern University of Science and Technology
Shenzhen 518055, China
E-mail: liuws@sustech.edu.cn
R. Shu, A. Elsukova, J. Lu, P. Å. Persson, J. Palisaitis, A.le Febvrier, P. Eklund
Thin Film Physics Division, Department of Physics Chemistry, and Biology (IFM), Linköping University, Linköping SE-581 83, Sweden
E-mail: per.eklund@liu.se
Prof. W. Q. Zhang
Department of Physics
Southern University of Science and Technology
Shenzhen 518055, China
O. Cojocaru-Mirédin, Y. Yu
I. Physikalisches Institut (IA)
RWTH Aachen University
Sommerfeldstraße14, 52074 Aachen, Germany
Email： yu@physik.rwth-aachen.de
W. S. Liu
Guangdong Provincial Key Laboratory of Functional Oxide Materials and Devices
Southern University of Science and Technology
Shenzhen 518055, Guangdong, China

The amorphous limit of lattice thermal conductivity

The amorphous limit of lattice thermal conductivity for $Mg_{3.2}Sb_{1.5}Bi_{0.5}$ was determined using the Cahill model [1]:

$$\kappa_{Lmin} = \left(\frac{\pi}{6}\right)^{1/3} k_B n^{2/3} \sum_1 v_i \left\{\frac{T}{\theta_i}\right\} \int_0^{\theta_i/T} \frac{x^3 e^x}{(e^x - 1)^2} d_x$$

where the sum is over the three sound modes with speeds of v_i, n represents the number density of atoms, and the cutoff frequency (θ_i) was determined as $\theta_i = v_i \left(\frac{h}{k_B}\right)\left(\frac{3n}{4\pi}\right)^{\frac{1}{3}}$.

The average phonon speed v was calculated based on the equation $\frac{1}{v^3} = \frac{2}{3v_t^3} + \frac{1}{3v_l^3}$, where the transverse and longitudinal wave velocities, v_t and v_l, were measured using a commercial resonant ultrasound spectroscopy (RUS) apparatus. The Lorenz number L was calculated based on the single parabolic band (SPB) model and assuming that acoustic phonon scattering dominated.

1. Microstructure of Ge-free $Mg_{3.2}Sb_{1.5}Bi_{0.5}$

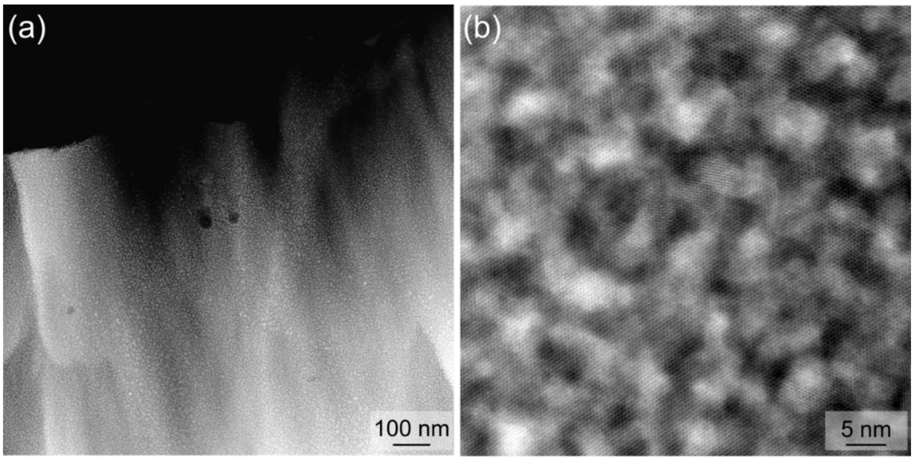

Figure S1. STEM images of Ge-free $Mg_{3.2}Sb_{1.5}Bi_{0.49}Te_{0.01}$ specimen. a) An overview STEM-HAADF image. b) HR-STEM HAADF image. Note that the bright and dark contrasts are due to composition fluctuations but not precipitates.

2. Phase composition and structure for n-type $Mg_{3.2}Sb_{1.5}Bi_{0.5}$ with different Ge-doping content

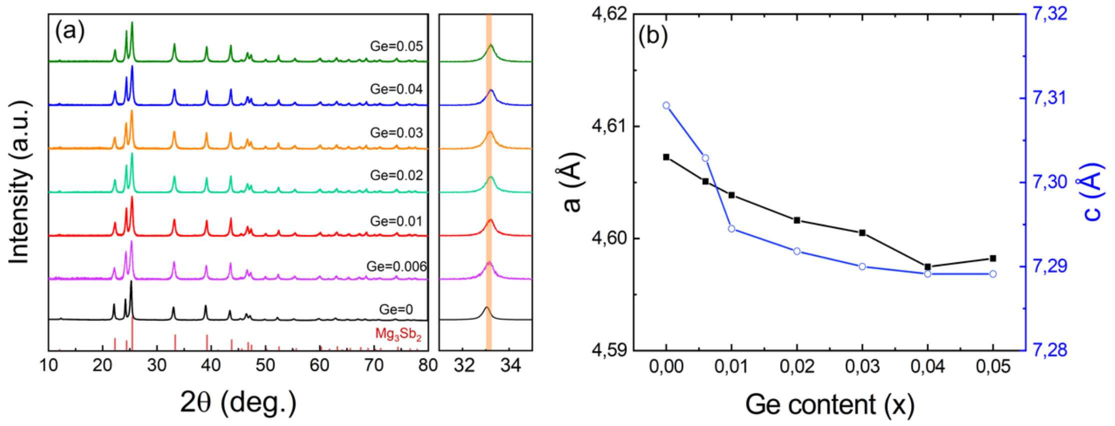

Figure S2. Structural characterization for n-type $Mg_{3.2}Sb_{1.5}Bi_{0.5}$ with different Ge-doping content. a) XRD patterns of as-fabricated $Mg_{3.2}Bi_{0.5}Sb_{1.49-2x}Te_{0.01+x}Ge_x$ specimens, no notable impurity phases were identified in this series of specimens. b) Lattice parameters of as-fabricated $Mg_{3.2}Bi_{0.5}Sb_{1.49-2x}Te_{0.01+x}Ge_x$ samples as a function of the Ge content x.

XRD patterns of as-fabricated $Mg_{3.2}Bi_{0.5}Sb_{1.49-2x}Te_{0.01+x}Ge_x$ samples with the nominal Ge compositions x =0, 0.006, 0.01, 0.02, 0.03, 0.04, 0.05 are displayed in Figure S2. All major reflections could be indexed to an inverse α-La_2O_3-type structure (space group, $P\bar{3}m1$) with no other impurity phase observed within the detection limits of powder XRD. The lattice parameters of $Mg_{3.2}Sb_{1.5}Bi_{0.5}$ solid solutions decreased with increasing Ge (x) and Te content. A trend is observed of decreasing lattice parameters calculated from the XRD patterns (Figure S2) and can be attributed to a reduction of the unit cell caused by Bi precipitates out from the $Mg_{3.2}Sb_{1.5}Bi_{0.5}$ solid solutions. This is consistent with the distance between (002) and (010) spots of Ge-0.05 sample in the corresponding SAED pattern (inset in Figure 2a in the main text) is shorter than that for the Ge-0.01 sample (Figure 1a in the main text).

3. Structure analysis of Bi- and Ge-rich precipitates and matrix observed in $Mg_{3.2}Sb_{1.39}Bi_{0.5}Te_{0.06}Ge_{0.05}$ specimen

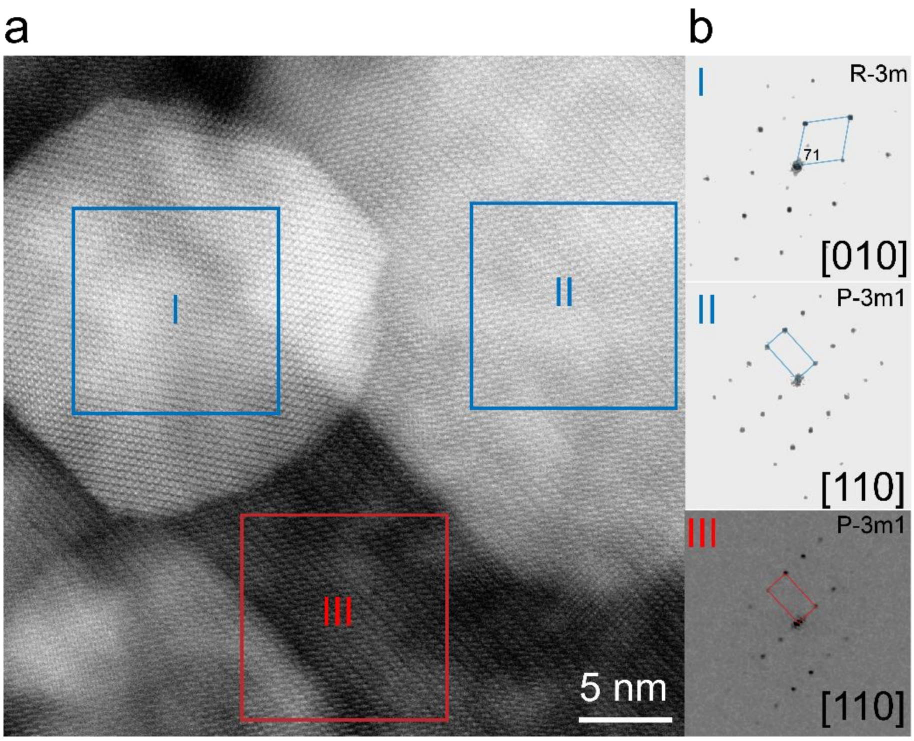

Figure S3. Identification of Bi-rich trigonal Mg_3Bi_2-type phase. a) HR-STEM-HAADF image of $Mg_{3.2}Sb_{1.39}Bi_{0.5}Te_{0.06}Ge_{0.05}$ specimen. b) FFT patterns of corresponding Bi-rich region I and II, and matrix region III.

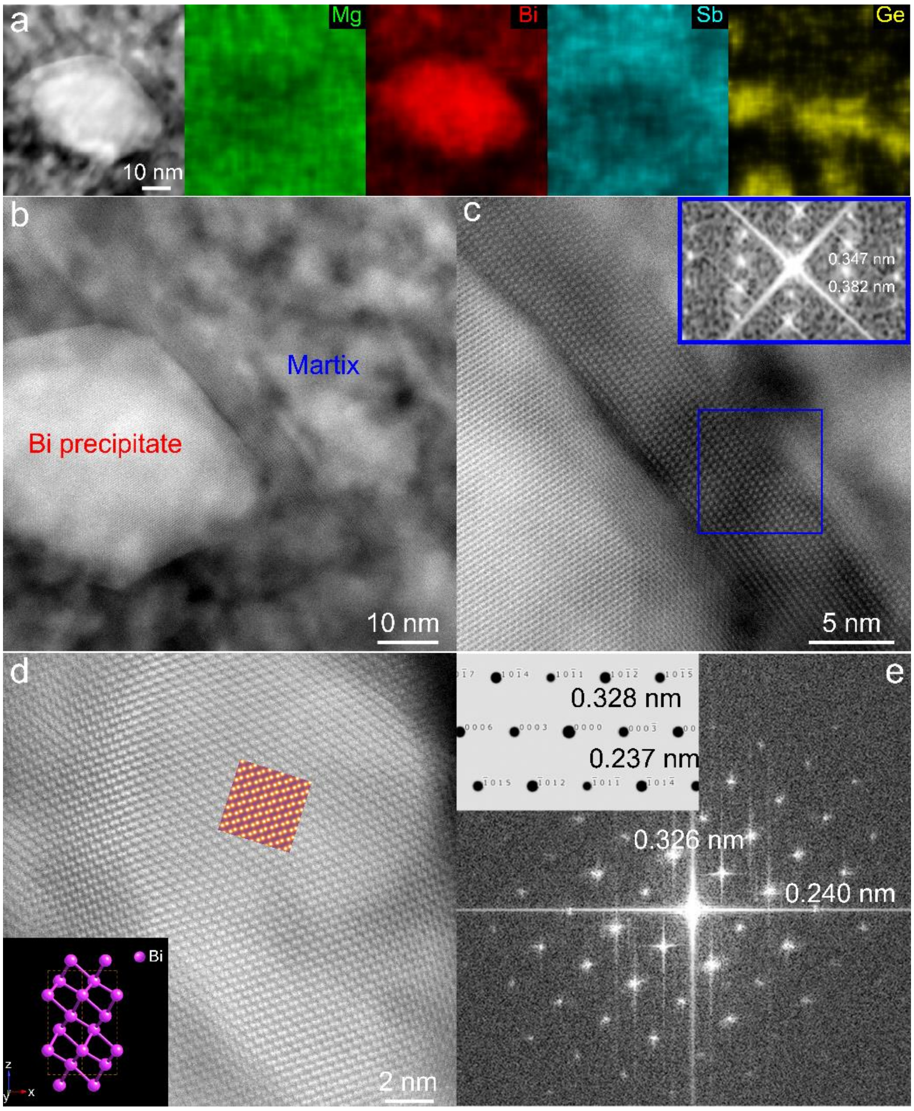

Figure S4. Structural characterization for Bi-rich precipitate of the $Mg_{3.2}Sb_{1.39}Bi_{0.5}Te_{0.06}Ge_{0.05}$ specimen. a) HAADF-STEM image of Bi-rich precipitate in $Mg_3Sb_{1.5}Bi_{0.5}$ matrix and corresponding Mg, Bi, Sb, and Ge elemental maps. b) HAADF-STEM image of Bi-rich precipitate and Mg-Sb-Bi matrix interface. c) High-resolution HAADF-STEM image of the precipitate-matrix interface. Inset: FFT pattern obtained from the region marked with blue square. d) High-resolution HAADF-STEM image of Bi-rich precipitate. Insets: crystal model of Bi (space group R-3m) viewed in [010] direction and corresponding simulated HAADF-STEM image. e) FFT pattern obtained from image d) Inset: Simulated diffraction pattern of Bi structure viewed in [010] direction.

We located Bi-rich precipitates by performing EDX elemental mapping (Figure S4a). Analysis of high resolution HAADF-STEM images of the precipitate (containing 61 at % of Bi) and its interface with matrix (Figure S4 b-e) showed that

the precipitate has the structure of rhombohedral Bi.

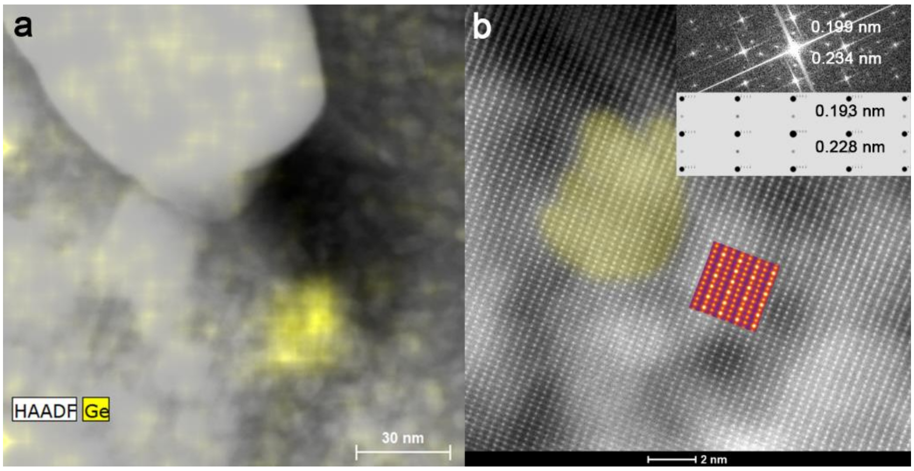

Figure S5. Structural characterization for Ge-rich precipitate of the $Mg_{3.2}Sb_{1.39}Bi_{0.5}Te_{0.06}Ge_{0.05}$ specimen. a) HAADF-STEM image with corresponding Ge elemental maps. b) High-resolution HAADF-STEM image of Ge-rich precipitate region. Inset on top, FFT pattern obtained from image b). Inset at the bottom, Simulated diffraction pattern of Mg_3Sb_2 (space group P-3m1) viewed in [120] direction and corresponding simulated HAADF-STEM image in the center.

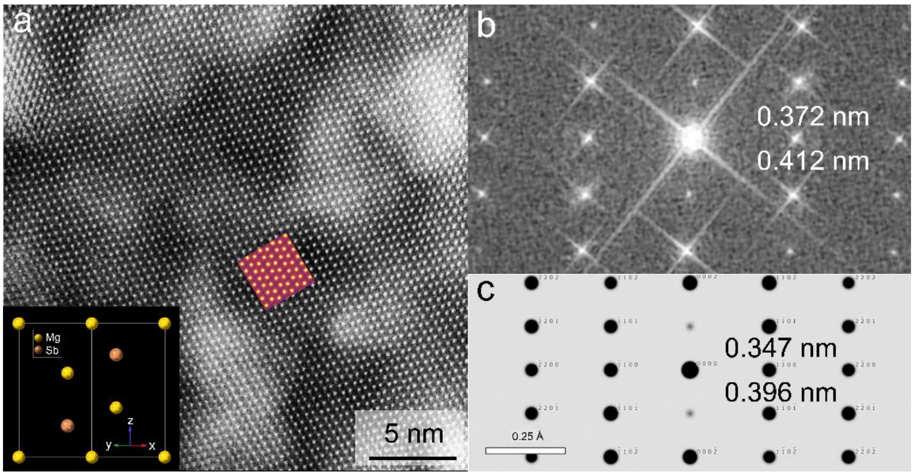

Figure S6. Structural characterization for the matrix of $Mg_{3.2}Sb_{1.39}Bi_{0.5}Te_{0.06}Ge_{0.05}$ specimen. a) High-resolution HAADF-STEM image of $Mg_3Sb_{1.5}Bi_{0.5}$ matrix. Lower inset: crystal model of Mg_3Sb_2 (space group P-3m1) viewed in [110] direction. Central inset: corresponding STEM simulation. b) FFT pattern obtained from image a. c) Simulated diffraction on Mg_3Sb_2 viewed in [110] direction.

The crystal structure of $Mg_3Sb_{1.5}Bi_{0.5}$ matrix for the $Mg_{3.2}Sb_{1.39}Bi_{0.5}Te_{0.06}Ge_{0.05}$ specimen was determined by comparing the contrast in HAADF-STEM image (Figure S6a) with STEM simulation (Figure 6a, central inset), and FFT pattern (Figure S6b) with the simulated diffraction pattern (Figure S6c) of the Mg_3Sb_2 P-3m1 structure viewed in [110] direction.

4. APT characterization on medium Ge-doping $Mg_{3.2}Bi_{0.5}Sb_{1.43}Te_{0.04}Ge_{0.03}$

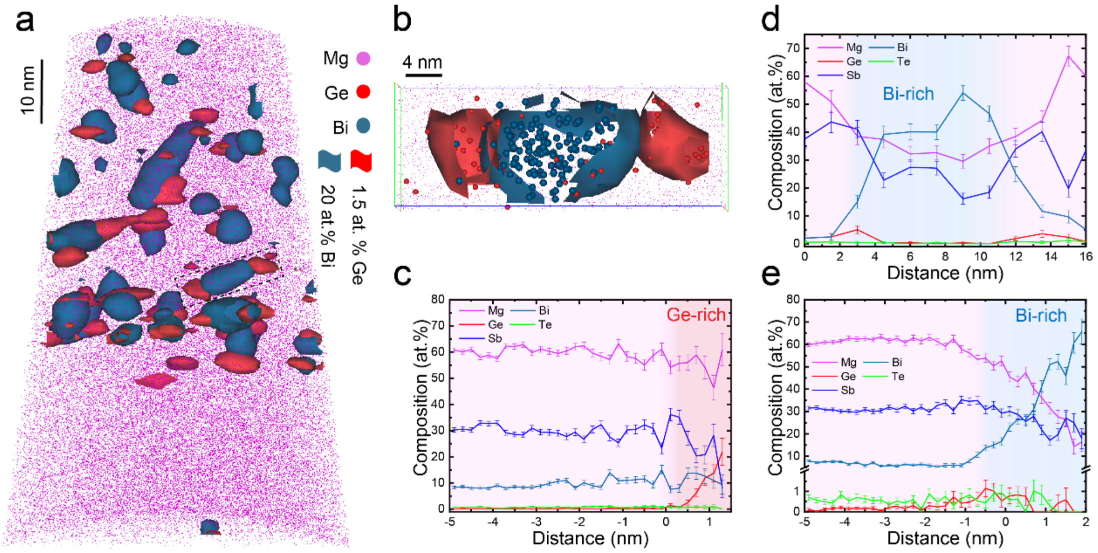

Figure S7. Three-dimensional elemental distribution analysis on nanostructures in the $Mg_{3.2}Bi_{0.5}Sb_{1.43}Te_{0.04}Ge_{0.03}$ specimen. a) APT reconstructions showing the distribution of elements (Mg, pink; Ge, red; Bi, teal); the Ge-rich and Bi-rich precipitates are depicted by iso-composition surfaces of 1.5 at.% Ge and 20 at.% Bi, respectively. Both precipitates connect side-by-side forming Janus precipitates. b) Close-up of a subregion taken from Figure S7a highlighting the 3D structure of Bi/Ge-rich Janus nanoprecipitates. c) composition proximity histogram calculated across the iso-composition surface of 1.5 at.% Ge showing the maximum Ge content at the precipitate core. d) 1D compositional profile along the horizontal direction of b showing the Bi-rich precipitate sandwiched by two Ge-rich precipitates. e) proximity histogram of 20 at.% Bi iso-surface showing the composition from the matrix to the Bi-rich precipitate core.

5. Comparison of thermoelectric performance

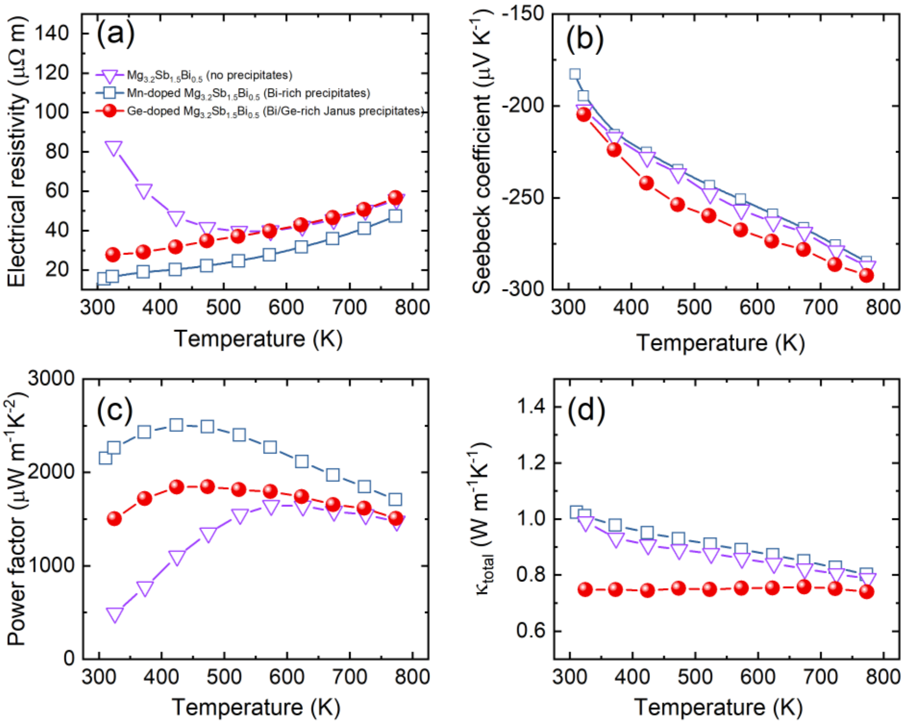

Figure S8. Temperature dependences of a) electrical resistivity, b) Seebeck coefficient, c) power factor, d) total thermal conductivity κ_{tot}, for dopant-free $Mg_{3.2}Sb_{1.5}Bi_{0.5}$ (no precipitates), $Mg_{3.15}Mn_{0.05}Sb_{1.5}Bi_{0.5}$ [single-type Bi-rich precipitates, ref.[2]] and $Mg_{3.2}Sb_{1.47}Bi_{0.5}Te_{0.02}Ge_{0.01}$ (Bi/Ge-rich Janus precipitates) in the direction perpendicular to the pressure applied to the samples during synthesis.

6. Precipitates-size effects on lattice thermal conductivity

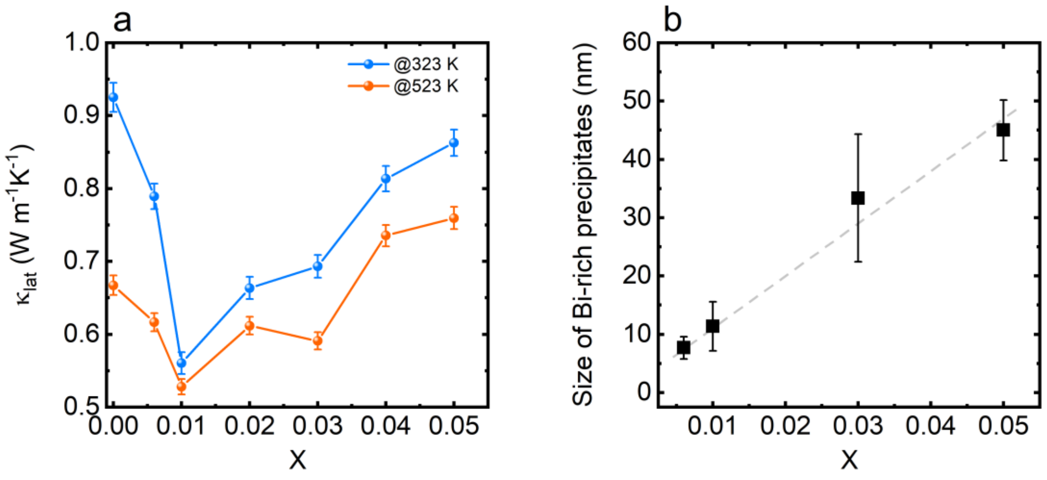

Figure S9. a) Lattice thermal conductivity κ_{lat} at 323 K and 523 K as a function of Ge dopant fraction x. b) The size of Bi-rich precipitate determined from APT and HRTEM images as a function of Ge dopant fraction x.

7. Thermoelectric performance overview for Ge-doped $Mg_{3.2}Sb_{1.5}Bi_{0.5}$

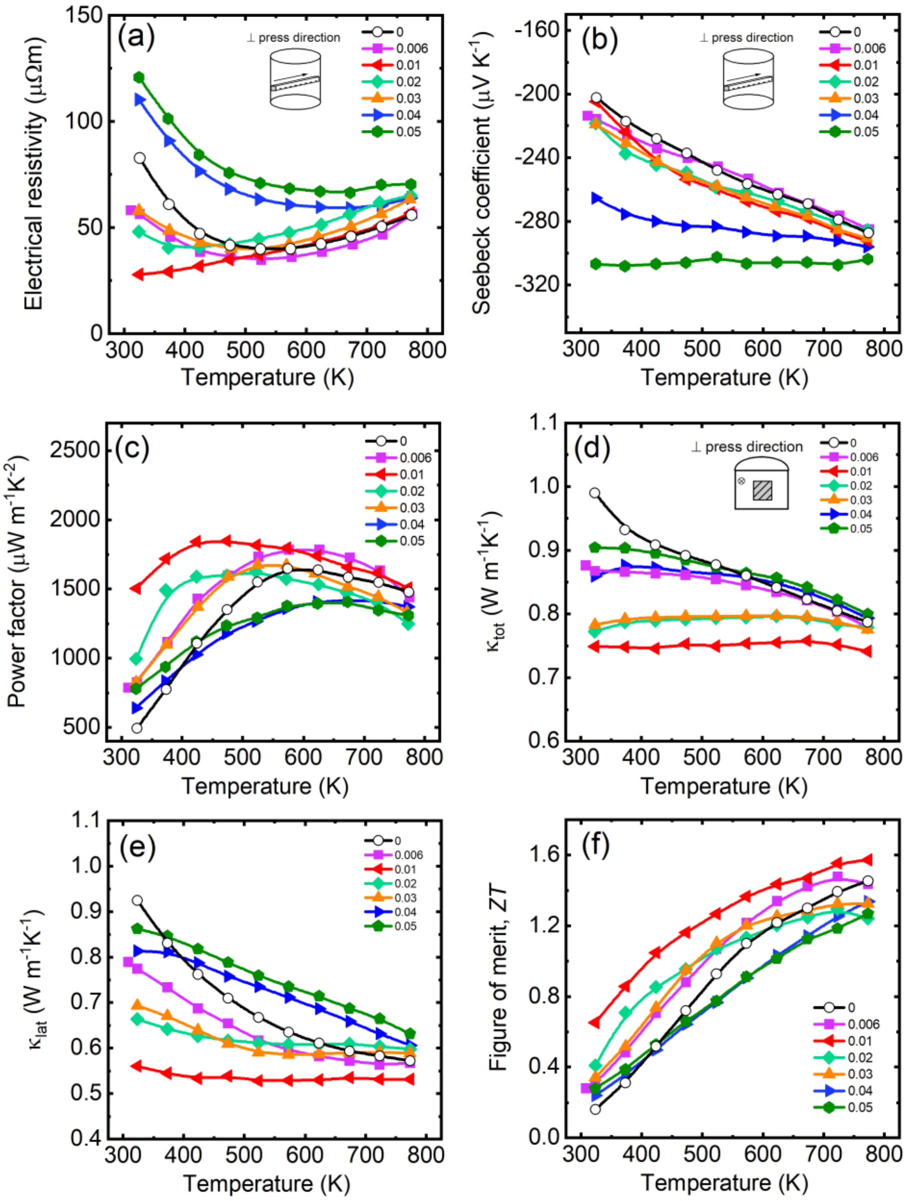

Figure S10. Electrical and thermal transport behavior in different dopant contents. Temperature dependences of a) electrical resistivity, b) Seebeck coefficient, c) power factor, d) total thermal conductivity κ_{tot}, e) lattice thermal conductivity κ_{lat} and f) ZT values for $Mg_{3.2}Sb_{1.49-2x}Bi_{0.5}Te_{0.01+x}Ge_x$ specimens (x=0-0.05) in direction perpendicular (⊥) to SPS press direction.

The electrical resistivity of the x=0 sample first decreases and then increases with temperature. This is a typical phenomenon observed in polycrystalline Mg_3Sb_2-based compounds, which can be attributed to the grain boundary potential barrier scattering of electrons.[3] By adding a slight content of Ge (x=0.006 and 0.01), the barrier height decreases

due to the segregation of Ge and Bi to grain boundaries. Yet, we found that the barrier height increases again with further increasing the content of Ge (Figure S10a), thus increasing the electrical resistivity, especially at low temperatures. Both STEM and APT proved that the size of nanoprecipitates increases with the Ge content. A larger particle can cause stronger charge carrier scattering at the interface and thus increase the electrical resistivity and the Seebeck coefficient due to potential energy filtering effect[4-6]. This explains the x=0.04 and x=0.05 samples showing the largest resistivity and absolute value of Seebeck coefficients. A suitable Ge content can compensate for the vacancy electron scattering in the pristine $Mg_{3.2}Sb_{1.5}Bi_{0.5}$ as reported in other transition-metal doped samples[5,6]. As a consequence, the x=0.01 sample shows the lowest electrical resistivity and the highest power factor (Figure S10). The extremely low thermal conductivity for the x=0.01 sample results from the phonon scattering of Janus particles and the size effect of nanoparticles, as discussed in the main text. Too large precipitates could not effectively scatter phonons due to the short phonon mean free path in Mg_3Sb_2. In contrast, the high intrinsic thermal conductivity of Bi and Ge could in turn increase the overall lattice thermal conductivity as observed in the high-content Ge samples. As a consequence, the optimized ZT values are obtained in the sample x=0.01. Note that the ZT values obtained in this work are not the highest reported in n-type Mg_3Sb_2 compounds. However, the Janus nanoprecipitation phenomenon observed in this work may be used to further enhance thermoelectric properties.

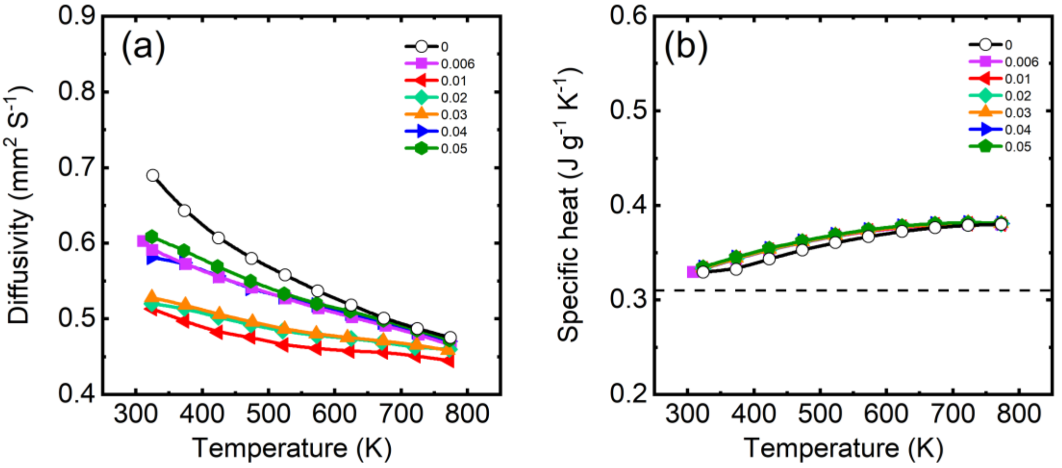

Figure S11. Thermal diffusivities and specific heat of $Mg_{3.2}Sb_{1.49\text{-}2x}Bi_{0.5}Te_{0.01+x}Ge_x$ specimens (x=0-0.05).

Table S1. Densities fof $Mg_{3.2}Sb_{1.49\text{-}2x}Bi_{0.5}Te_{0.01+x}Ge_x$ specimens (x=0-0.05).

Specimen (x)	0	0.006	0.01	0.02	0.03	0.04	0.05
Density (± 0.01g cm^{-3})	4.38	4.41	4.43	4.45	4.44	4.42	4.44

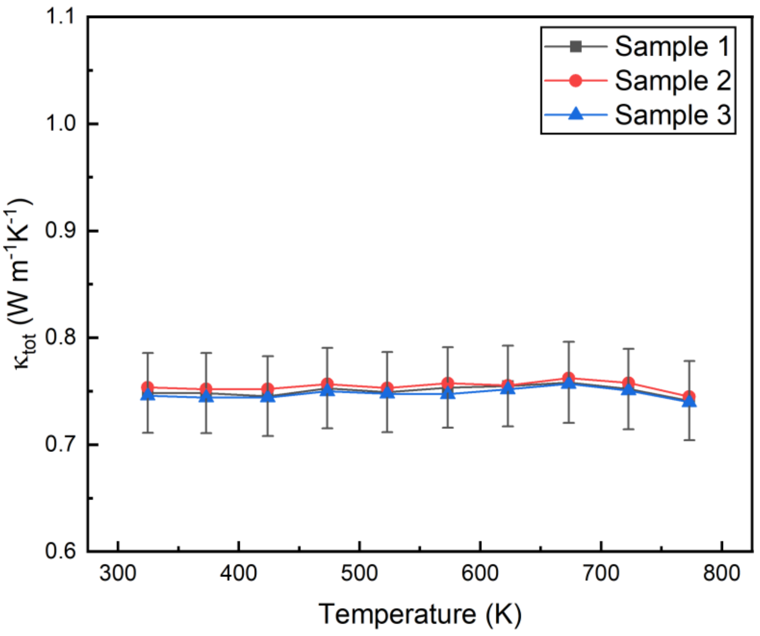

Figure S12. Thermal conductivity of $Mg_{3.2}Sb_{1.47}Bi_{0.5}Te_{0.02}Ge_{0.01}$.

8. Phonon scattering of the Janus nanoparticles

The Callaway model [6] is a well-known theoretical basis to interpret the temperature dependent lattice thermal conductivity (κ_{lat}). Here, we will use the same basis to consider the effect of the Janus nanoprecipitates on the thermal conductivity according to the Matthiessen's rule.[7]

$$\kappa_{lat} = \frac{k_B}{2\pi^2 \upsilon} \int_0^{\frac{k_B \theta_D}{\hbar}} \tau_{tot} \left(\frac{\hbar \omega}{k_B T} \right)^2 \frac{\exp(\hbar \omega / k_B T)}{\left[\exp(\hbar \omega / k_B T) - 1\right]^2} \omega^2 d\omega \qquad \text{(S1)}$$

$$\tau_{tot}^{-1} = \tau_U^{-1} + \tau_{PD}^{-1} + \tau_B^{-1} + \tau_{NP}^{-1} + \tau_{Janus}^{-1} \qquad \text{(S2)}$$

$$\tau_{PD}^{-1} = A \cdot \omega^4 \qquad \text{(S3)}$$

$$\tau_U^{-1} = BT \cdot \omega^2 \qquad \text{(S4)}$$

$$\tau_B^{-1} = \frac{\upsilon}{l} \qquad \text{(S5)}$$

where the frequency dependent relaxation time τ_{tot}, τ_U, τ_{ph}, τ_B, τ_{NP}, and τ_{Janus},are the combined relaxation time, the partial contribution due to Umklapp phonon-phonon scattering, phonon-point defects, phonon-boundary, nanoparticles scattering, and phonon-Janus particles, respectively. The phonon scattering of the Janus particles could be considered as a combination of an average effect of the whole particle and partial contribution of the internal sub-nanoparticle, as follows:

$$\frac{1}{\tau_{jnp}} = \frac{1}{\tau_{\langle jnp \rangle}} + \sum_i \frac{1}{\tau_{np-i}} \qquad \text{(S6)}$$

For the phonon scattering of nanoparticles embedded in an alloy, Majumdar et al.[8] have proposed a cross-section combination of the Rayleigh limit (σ_l) and Geometric limit (σ_s).

$$\frac{1}{\tau_{np}} = \upsilon \rho \left(\frac{1}{\sigma_s} + \frac{1}{\sigma_l} \right)^{-1} \qquad \text{(S7)}$$

$$\sigma_s = 2\pi R^2 \tag{S8}$$

$$\sigma_l = \frac{4}{9}\pi R^2 \cdot \left(\frac{\Delta D}{D_0}\right)^2 \left(\frac{\omega R}{\upsilon}\right)^4 \tag{S9}$$

Where ρ is the particles concentration, ΔD is the density difference between the nanoparticle and the matrix, D_0 is the density of the matrix, ω is the angular frequency, v is the average phonon speed, and R is the average radius of nanoparticles.

Considering our matrix is a heavily doped system with spherical nanoparticles, we only consider the Rayleigh limit (σ_l), the Eq. (S7-9) could be

$$\frac{1}{\tau_{np}} = \frac{\upsilon}{3}\frac{f_{np}}{R}\left(\frac{\Delta D}{D_0}\right)^2 \left(\frac{\omega}{\upsilon}R\right)^4 \tag{S10a}$$

Or considering both the Rayleigh limit (σ_l) and Geometric limit (σ_s).

$$\frac{1}{\tau_{np}} = \frac{\upsilon}{3}\frac{f_{np}}{R}\left(\frac{2}{9} + \frac{1}{\left(\frac{\Delta D}{D_0}\right)^2 \left(\frac{\omega}{\upsilon}R\right)^4}\right)^{-1} \tag{S10b}$$

Now we consider a general case, i.e. a given volume fraction of the Janus nanoparticle f_{jnp} with an average particle size of $2R_{\text{jnp}}$, the average density of D_{jnp}, which contains i sub-nanoparticles with an average particle size of $2R_{\text{i-snp}}$ and the average density of $D_{\text{i-snp}}$. Then the Eq. S6 could be further expressed as follows:

$$\frac{1}{\tau_{jnp}} = \frac{1}{\tau_{\langle jnp\rangle}} + \sum_i \frac{1}{\tau_{np-1}} \tag{S11}$$

$$\frac{1}{\tau_{jnp}} = \frac{\upsilon}{3}\frac{f_{\langle jnp\rangle}}{R_{\langle jnp\rangle}}\left(\frac{D_{\langle jnp\rangle} - D_0}{D_0}\right)^2 \left(\frac{\omega}{\upsilon}R_{\langle jnp\rangle}\right)^4 + \sum_i \frac{1}{3(m\upsilon)^3}\frac{f_{i-np}}{R_{i-np}}\left(\frac{D_{i-np} - D_{\langle jnp\rangle}}{D_{\langle jnp\rangle}}\right)^2 \left(\omega R_{i-np}\right)^4$$

$$f_{\langle jnp \rangle} = \sum_i f_{i-np}$$

where a correction factor *m* is introduced to interpret partial contribution of the internal sub-nanoparticle of Janus nanoparticle, and relative with average phonon speed υ. Next, we input equation S11 back to S1 and S2, and introduce the parameter $x = \hbar\omega / k_B T$, and applied to our case.

$$\kappa_{lat} = \frac{k_B}{2\pi^2 \upsilon_s} \left(\frac{k_B T}{\hbar} \right)^3 \int_0^{k\theta_D} \tau_{tot} \frac{x^4 e^x}{\left(e^x - 1\right)^2} dx$$

$$\frac{1}{\tau_{tot}} = A' \cdot T^4 \cdot x^4 + B' \cdot T^3 \cdot x^2 + \frac{\upsilon}{l}$$

$$+ C' f_{\langle jnp \rangle} \left(\frac{D_{\langle jnp \rangle} - D_0}{D_0} \right)^2 T^4 R^3_{\langle jnp \rangle} x^4 + \sum_i C' f_{i-np} \left(\frac{D_{i-np} - D_{\langle jnp \rangle}}{D_{\langle jnp \rangle}} \right)^2 T^4 R^3_{i-np} x^4$$

$$A' = A \left(\frac{k_B}{\hbar} \right)^4$$

$$B' = B \left(\frac{k_B}{\hbar} \right)^2$$

$$C' = \frac{1}{3\left(m\upsilon\right)^3} \left(\frac{k_B}{\hbar} \right)^4$$

Or considering both the Rayleigh limit (σ_l) and Geometric limit (σ_s) in the $\tau_{<jnp>}$

$$\frac{1}{\tau_{tot}} = A^{'} \cdot T^{4} \cdot x^{4} + B^{'} \cdot T^{3} \cdot x^{2} + \frac{\upsilon}{l}$$

$$+ C \frac{f_{\langle jnp \rangle}}{R_{\langle jnp \rangle}} \left(\frac{2}{9} \left(\frac{\hbar}{k_B} \right)^{4} + \frac{1}{\left(\frac{D_{\langle jnp \rangle} - D_0}{D_0} \right)^{2} T^{4} R^{4}_{\langle jnp \rangle} x^{4}} \right)^{-1} + \sum_i C^{'} \frac{f_{i-np}}{R_{i-np}} \left(\frac{D_{i-np} - D_{\langle jnp \rangle}}{D_{\langle jnp \rangle}} \right)^{2} T^{4} R^{4}_{i-np} x^{4}$$

$$A^{'} = A \left(\frac{k_B}{\hbar} \right)^{4}$$

$$B^{'} = B \left(\frac{k_B}{\hbar} \right)^{2}$$

$$C = \frac{1}{3\upsilon^{3}} \left(\frac{k_B}{\hbar} \right)^{4}$$

$$C^{'} = \frac{1}{3(m\upsilon)^{3}} \left(\frac{k_B}{\hbar} \right)^{4}$$

Table S2. The parameters used for theoretical calculations of the lattice thermal conductivity of $Mg_{3.2}Sb_{1.47}Bi_{0.5}Te_{0.02}Ge_{0.01}$ with Janus nanoprecipitates. The experimental parameters are from the STEM and APT investigations.

Parameter	Symbol	Value	Units
**Experiment parameter**			
Average phonon speed		1800	m/s
Debye temperature	θ_D	179.12	K
Atoms per unit cell	N	5	-
Unit cell volume	V_Janus	1.34E-28	m^3
Lattice constants	c_Janus	7.29	Å
	a_Janus	4.60	Å
Volume fraction of Janus nanoparticle	f_{jnp}	3.45	vol%
Average particle size of Janus nanoparticle	$2R_{jnp}$	1.48E-08	m
Average density of Janus nanoparticle	D_{jnp}	7.46	g/cm^3
Matrix density	D_0	4.43	g/cm^3
Volume fraction of Bi-rich precipitate	f_{Bi}	1.65	vol%
Average particle size of Bi-rich precipitate	$2R_{Bi}$	2.00E-08	m
Average density of Bi-rich precipitate	D_{Bi}	9.80	g/cm^3
Volume fraction of Ge-rich precipitate	f_{Ge}	1.80	vol%
Average particle size of Ge-rich precipitate	$2R_{Ge}$	1.00E-08	m
Average density of Ge-rich precipitates	D_{Ge}	5.32	g/cm^3
**Constant parameter**			
Planck constant	h	6.63E-34	Js
Boltzmann constant	k_B	1.38E-23	J/K
**Fitting parameter**			
Point defect scattering coefficient	A	1.00E-40	S^2
Umklapp phonon scattering coefficient	B	1.05E-17	s/K
Distance between Janus particles	l	5.00E-06	m
Individual particle sound velocity adjustment coefficient	n	16	-

References

1. Cahill, D. G., Watson, S. K. & Pohl, R. O. Lower limit to the thermal conductivity of disordered crystals. *Phys. Rev. B* **46**, 6131–6140 (1992).
2. Chen, X. *et al.* Extraordinary thermoelectric performance in n-type manganese doped Mg_3Sb_2 Zintl: high band degeneracy, tuned carrier scattering mechanism and hierarchical microstructure. *Nano Energy* **52**, 246–255 (2018).
3. A. Li, C. Fu, X. Zhao, T. Zhu, High-performance $Mg_3Sb_{2-x}Bi_x$ thermoelectrics: Progress and perspective. *Research*. **2020**, 1–22 (2020).
4. Mao, J. *et al.* Manipulation of ionized impurity scattering for achieving high thermoelectric performance in n-type Mg_3Sb_2-based materials. *Proc. Natl. Acad. Sci.* 201711725 (2017).
5. Shuai, J. *et al.* Tuning the carrier scattering mechanism to effectively improve the thermoelectric properties. *Energy Environ. Sci.* **10**, 799–807 (2017).
6. J. Callaway, H.C. von Baeyer, *Phys. Rev.*, **120**, 1149-1154, (1960).
7. Z. W. Chen, Z. Z. Jian, W. Li, Y. J. Chang, B. H. Ge, R. Hanus, J. Yang, Y. Chen, M. X. Huang, G. J. Snyder, Y. Z. Pei, Adv. Mater. **29**, 1606768, (2017).
8. W. Kim, A. Majumdar, J. Appl. Phys., **99**, 084306, (2006).
